# Psoas muscle volume as a diagnostic indicator for sarcopenia: criteria development and comparison with traditional diagnostic approaches

**DOI:** 10.1007/s40520-026-03365-9

**Published:** 2026-03-15

**Authors:** Woorim Choi, Chul-Ho Kim, Ji Wan Kim

**Affiliations:** 1 Biomedical Research Center, Asan Medical Center, Seoul, Republic of Korea; 2 Department of Orthopedic Surgery, Asan Medical Center, Seoul, Republic of Korea; 3 University of Ulsan College of Medicine, Seoul, Republic of Korea

**Keywords:** Sarcopenia criteria, Psoas muscle volume, Psoas index, ASM index

## Abstract

**Background:**

Sarcopenia significantly impacts quality of life and increases the risk for various health issues. This study aims to establish a new, effective diagnostic criterion for sarcopenia based on psoas muscle volume (PV) using computed tomography (CT).

**Aims:**

We analyzed the population distribution of psoas muscle volume (PV), assessed its correlation with ASM indices, and developed CT-based diagnostic criteria for sarcopenia.

**Methods:**

A total of 3,999 adults (2,085 men, 1,914 women; aged 22–89) who underwent abdominal CT and bioimpedance analysis (BIA) were included. Psoas muscle volume was automatically segmented using a deep-learning algorithm. Correlations with ASM indices were evaluated, and diagnostic criteria were established using (1) linear regression, (2) ROC analysis, and (3) T-score analysis referencing young, non-sarcopenic adults.

**Results:**

The distribution of PV indices peaked in the 30s and declined with age, more sharply in men. PV showed strong correlations with ASM indices, particularly the PV/BMI index, which demonstrated the highest diagnostic accuracy. T-score adjustment to -2.0 better matched known prevalence rates.

**Discussion:**

This study proposes CT-based diagnostic criteria for sarcopenia using psoas muscle volume, demonstrating strong correlation with established indices. These findings support opportunistic screening via CT, offering a practical, population-wide tool for early sarcopenia detection.

**Conclusions:**

This study uncovers the distribution of PV across age groups, noting a significant decline from the 30s to the 70s. This advancement in the objective diagnosis of sarcopenia via imaging positions abdomen CT scans as a potential diagnostic screening tool for low muscle mass compatible with sarcopenia.

## Introduction

Sarcopenia is defined by the European Working Group on Sarcopenia in Older People (EWGSOP2) as low muscle strength, reduced muscle mass or quality, and poor physical performance, the latter reflecting severity [1]. This musculoskeletal condition impairs mobility and increases fall risk, significantly reducing quality of life. It frequently coexists with fractures, diabetes, periodontitis, and cardiovascular diseases, compounding health risks [2–5]. Therefore, accurate diagnosis and early prevention are increasingly important. Current diagnostic tools include dual-energy X-ray absorptiometry (DXA), magnetic resonance imaging (MRI), and CT [6]. However, DXA lacks accuracy, MRI is costly, and CT involves radiation exposure. This underscores the need for safer, effective alternatives. Our study proposes reusing CT scans from comprehensive health screening visits for opportunistic screening of low muscle mass characteristic of sarcopenia.

Bioelectrical impedance analysis (BIA) is also widely used in clinical practice and epidemiologic studies because it is rapid, noninvasive, and accessible for estimating appendicular skeletal muscle mass (ASM). However, BIA-derived estimates can be affected by measurement conditions and device-/equation-specific variability; in particular, changes in hydration status and recent food/fluid intake may influence impedance-based body composition estimates, and BIA is not recommended in some individuals (e.g., those with implanted electronic devices). Therefore, in the present study, we used BIA-derived ASM indices as established reference definitions of low muscle quantity (Table 1), while clearly reporting the BIA device (InBody) and standardized measurement conditions in the Methods to improve transparency and reproducibility.

**Table 1:** The diagnostic criteria of PV indices and its calculated prevalence according to three pivotal analysis

From Reference				Correlation analysis	ROC Analysis		T-score < -2.5	T-score < -2.0
Reference study	ASM index	Criteria (prevalence)	PV Indices	Determined criteria (prevalence)	Determined criteria (prevalence)	AUC	Determined criteria (prevalence)	Determined criteria (prevalence)
A. Male								
Studenski et al. [17]	ASM	20 kg (16%)	PV	308 cm^3^ (11%)	276 cm^3^ (5%)	0.911	296 cm^3^ (8%)	340 cm^3^ (18%)
Lim et al. [18]	wSMI	29.9% (19%)	PV/Weight	3.90 cm^3^/kg (2%)	4.04 cm^3^/kg (2%)	0.732	4.16 cm^3^/kg (4%)	4.63 cm^3^/kg (10%)
Kim et al. [19]	ASM/ BMI	0.90 m^2^ (36%)	PV/BMI	15.4 cm^3^*m^2^/kg (28%)	12.0 cm^3^*m^2^/kg (5%)	0.797	12.4 cm^3^*m^2^/kg (7%)	13.9 cm^3^*m^2^/kg (15%)
Chen et al. [20]	hSMI	7.0 kg/m^2^ (12%)	PV/Height^2^	96.2 cm^3^/m^2^ (8%)	97.0 cm^3^/m^2^ (4%)	0.882	100.0 cm^3^/m^2^ (5%)	113.8 cm^3^/m^2^ (13%)
B. Female								
Studenski et al. [17]	ASM	15 kg (42%)	PV	223 cm^3^ (42%)	173 cm^3^ (8%)	0.820	148 cm^3^ (3%)	173 cm^3^ (8%)
Lim et al. [18]	wSMI	25.1% (23%)	PV/Weight	3.22 cm^3^/kg (10%)	2.67 cm^3^/kg (2%)	0.817	2.87 cm^3^/kg (4%)	3.28 cm^3^/kg (12%)
Kim et al. [19]	ASM/ BMI	0.63 m^2^ (32%)	PV/BMI	8.93 cm^3^*m^2^/kg (27%)	6.85 cm^3^*m^2^/kg (5%)	0.881	7.21 cm^3^*m^2^/kg (7%)	8.37 cm^3^*m^2^/kg (19%)
Chen et al. [20]	hSMI	5.7 kg/m^2^ (23%)	PV/Height^2^	71.8 cm^3^/m^2^ (4%)	64.8 cm^3^/m^2^ (3%)	0.754	58.2 cm^3^/m^2^ (1%)	67.0 cm^3^/m^2^ (4%)

Based on reference values determined using ASM indices (ASM, wSMI, AASM/BMI, hSMI), we calculated the prevalence rates of our corresponding PV indices. The corresponding PV indices were determined using the following methods: correlational analysis, ROC, and T-score. The diagnostic criteria value and prevalence for the method yielding the closest match for each index (or row) is highlighted. ASM; Appendicular Skeletal Muscle, BMI; Body Mass Index

The psoas muscle is a deep core muscle that correlates with muscle mass and is a marker used to quantify muscle mass [7, 8]. The psoas muscle is commonly captured in various CT types (abdomen, pelvis, angiography, etc.) and has been used in previous sarcopenia studies via measures like thickness [9], volume [10], or area/height² [11]. While many studies focus on L3–L4 slices [12–14], standardized diagnostic criteria are lacking. Recent evidence shows muscle volume may be a more accurate indicator [15]. Our dataset does not include muscle strength or physical performance measures. The present work focuses on identifying individuals with low muscle quantity compatible with sarcopenia by developing CT-derived psoas muscle volume (PV) indices and cut-offs anchored to established low muscle mass definitions (ASM-based indices). This study aims to examine population-based PV distribution, compare it with ASM indices, and propose diagnostic criteria for sarcopenia based on PV.

## Materials and methods

### Study population

This cross-sectional study included 3,999 participants, aged 22 to 89, who visited Asan Medical Center for comprehensive health screening visits involving CT scans and body composition measurements from 2011 to 2022. From a larger pool of 12,000 candidates, participants were selected to represent different age groups. Specifically, the study included the maximum number of available individuals in their 20–30s and 70–80s. For those in the age range of 40–60s, around 400 subjects were randomly chosen for each age group, with a balanced representation of both genders.

All subjects underwent abdomen and pelvis CT imaging, and BIA as part of the health screening. Participants were excluded if they had factors known to compromise the validity of BIA, including limb amputation, metal prostheses, or implanted electronic devices. To standardize BIA measurements, all examinees were instructed to refrain from food and water intake for at least 2 h prior to assessment and to remove all metallic items on their body at the time of measurement. The study was approved by the Institutional Review Board (IRB No. 2021 − 0634) and complied with the 1964 Declaration of Helsinki.

### Anthropometric and body composition measurement

The subjects’ body composition was measured using the BIA method using Inbody (InBody Co., Ltd., Seoul, Republic of Korea). After measuring the right arm, left arm, trunk, right leg, and left leg separately, the ASM value was calculated by adding the muscle mass values ​​of the arms and legs. Skeletal muscle mass (SMM), which means total weight of ASM and trunk muscle, was measured. Body mass index (BMI) was calculated using the measured weight and height. ASM indices included ASM/height^2^, ASM/weight, ASM/BMI, ASM) and SMM indices (SMM, SMM/BMI, SMM/height^2^).

### Measurement of PV

All subjects APCT was taken using Somatom Definition (Siemens Healthineers, Erlangen, Germany), Discovery CT750 HD (GE Healthcare, Milwaukee, WI, US), or LightSpeed VCT scanner (GE Healthcare, Milwaukee, WI, US), and imaging was performed with the following parameter values: 120 kVp; automated dose modulation (CareDose 4D, Siemens Healthineers; automA and smartmA, GE Healthcare); matrix 512 × 512; collimation of 0.625 mm, slice thickness 5 mm.

Using CT images and the developed AVIEW software (Coreline soft, Ltd., Seoul, Republic of Korea), the psoas muscle area was automatically segmented and the PV value was quantified. The segmentation algorithm used the nn-Unet method. Separate from the dataset used in this paper, the above algorithm was trained using a training set (320 people), and sufficient reliability was secured by evaluating it through a validation set (200 people) [16].

The volume of the psoas muscle was adjusted to the PV index: height square (PV/height^2^), weight (PV/weight), body mass index (PV/BMI).

### Statistical analysis

We used unequal variance independent t-tests with Bonferroni correction for analyzing differences between consecutive age groups.

To establish diagnostic criteria for the PV index, we performed three pivotal statistical analyses: (i) correlation analysis, (ii) Receiver Operating Characteristic (ROC) analysis, and (iii) T-score analysis, utilizing the ASM index criteria currently employed for sarcopenia diagnosis.

We assessed the correlation between PV indices and previously established ASM indices. By utilizing Pearson’s correlation coefficient (r), we identified significant correlations (p-value less than 0.05) and pinpointed pairs of indices with high correlation as potential markers for diagnosing sarcopenia.

The diagnostic performance was determined by calculating the area under the curve (AUC) in ROC, and we established cutoff values for diagnosing sarcopenia based on achieving maximum specificity and sensitivity. This involved comparing the PV indices against established ASM indices as a reference.

The T-score for PV indices was established using data from the young adult demographic, specifically individuals aged 20–39. This age group’s PV, deemed as the normal baseline without indications of sarcopenia, provided the standard for reference. We calculated T-scores based on this group’s mean and standard deviation. A T-score threshold of -2.0 / -2.5 or lower was used to diagnose sarcopenia, reflecting a method akin to that employed in diagnosing osteoporosis through bone density measurements, but adapted for the evaluation of PV indices in sarcopenia.

$$T-score=\frac{Measurementvalue-Youngadult(ages20-39)mean}{Youngadult\left(ages20-39\right)standarddeviation}$$

## Results

### Distribution of Psoas indices

**Fig. 1:**
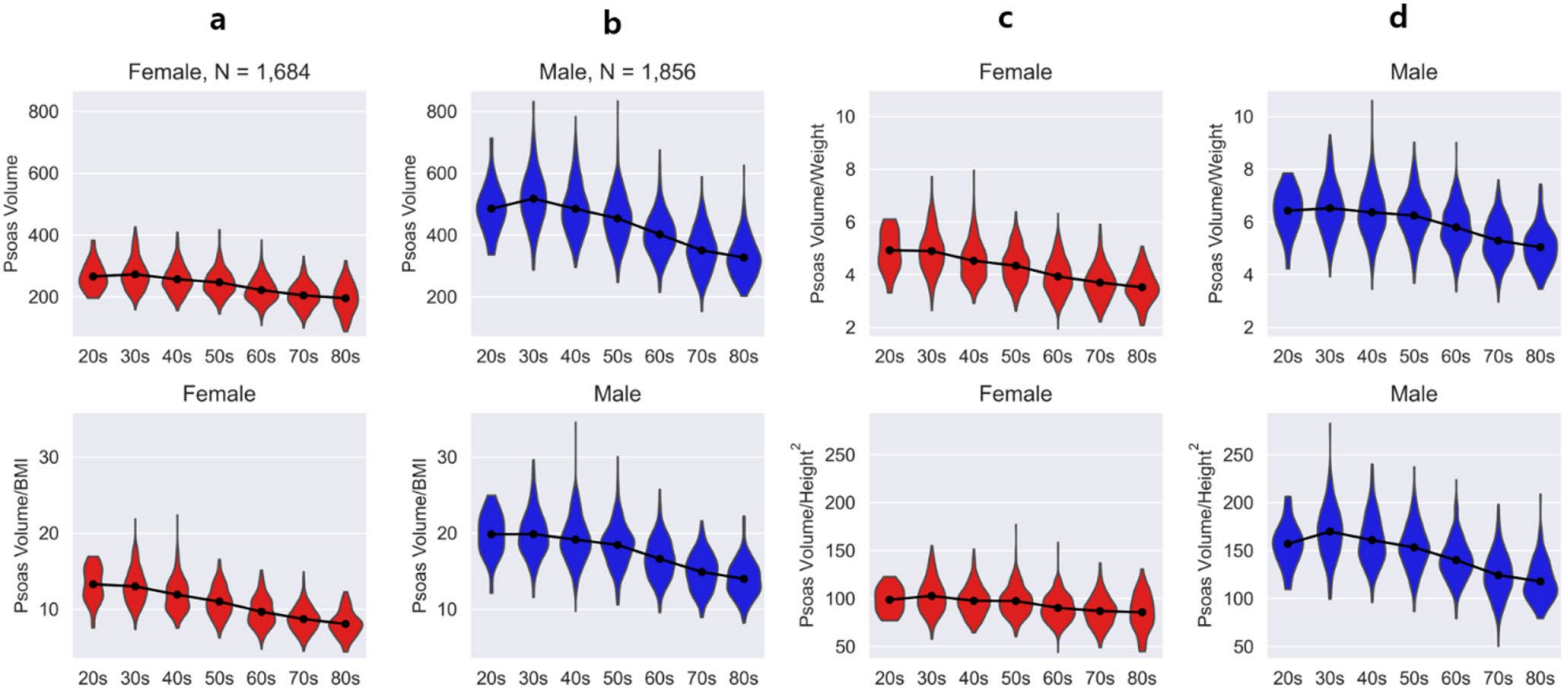
—Violin plot distribution of the psoas muscle volume indices by age group: (**a**) psoas muscle volume, (**b**) psoas muscle volume/weight, (**c**) psoas muscle volume/BMI, and (**d**) psoas muscle volume/height^2^ (*p < 0.05). BMI; Body Mass Index

The distribution of the four PV indices: (i) PV, (ii) PV/weight x 100 (%), (iii) PV/BMI, and (iv) PV/height^2^, is depicted in Fig. 1, categorized by gender and age.

#### Mean value comparison

Table 1 presents the average values of PV indices by gender and age, revealing higher indices in males than in females. A notable peak in these indices is observed in both genders during the 30s, followed by a gradual decline.

In the male results, PV indices exhibit a peak in the 30s and gradually decrease from the 40s onwards. Similarly, in females, there is also a peak in the 30s, followed by a gradual decline.

In female participants, we observed a similar pattern in most PV indices. These indices generally peaked during the 30s, and then consistently showed a decline starting from the 40s. Notably, the average value for the PV/weight index in women in their 30s was lower compared to that in their 20s. This trend of a gradual decrease in PV indices from the 40s onward across all age groups was a significant finding in our study.

#### Comparison of PV indices between adjacent age groups

Figure 1 includes the results of the t-test between adjacent age groups. Significant differences in various PV indices are observed across different age ranges for both genders.

There were no significant differences in any PV indices between the women in their 20s and 30s. However, from the 30s to the 70s, notable differences emerged in indices such as PV (*p* < 0.001), PV/weight (*p* < 0.001), and PV/BMI (*p* < 0.001). Particularly for PV/BMI, this trend of significant differences extended up to the 80s (*p* = 0.003). There were no significant differences in PV/height^2^ between the 40s and 50s age groups.

In male participants, our analysis revealed no significant differences in most PV indices between those in their 20s and 30s except for PV/height^2^ (*p* = 0.008). However, significant variations were found in the other psoas index: PV, PV/BMI, and PV/height^2^, from the 30s to the 80s (*p* < 0.001). For PV/weight, significant differences were observed only from the 50s to the 80s (*p* < 0.001).

In summary, a significant decrease in most PV indices from the 30s to 70s, with some exceptions noted in specific age groups and indices.

### Correlation analysis between ASM/SMM indices and PV indices

**Fig. 2:**
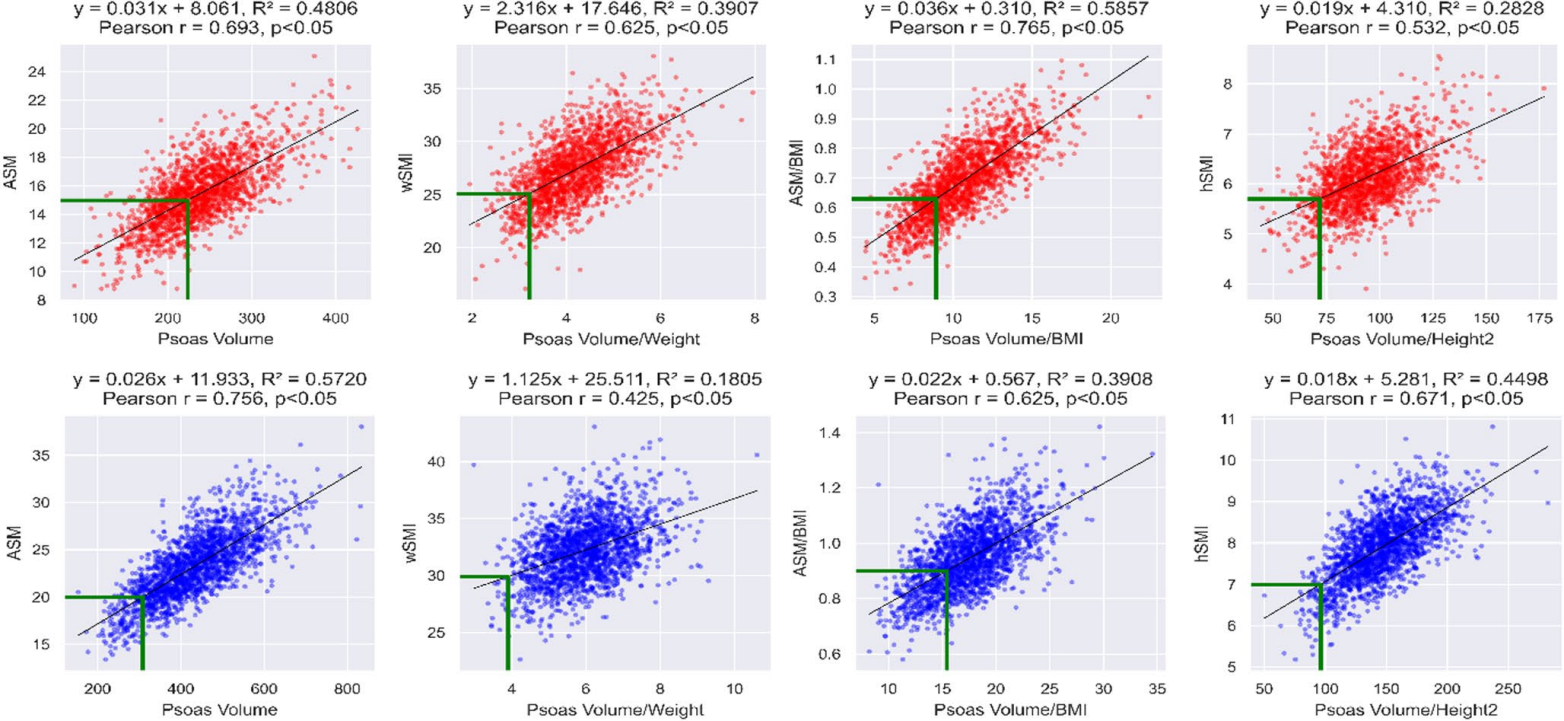
—Correlation between Psoas muscle volume indices and ASM indices (female - red, male - blue). ASM; Appendicular Skeletal Muscle, BMI; Body Mass Index

**Fig. 3:**
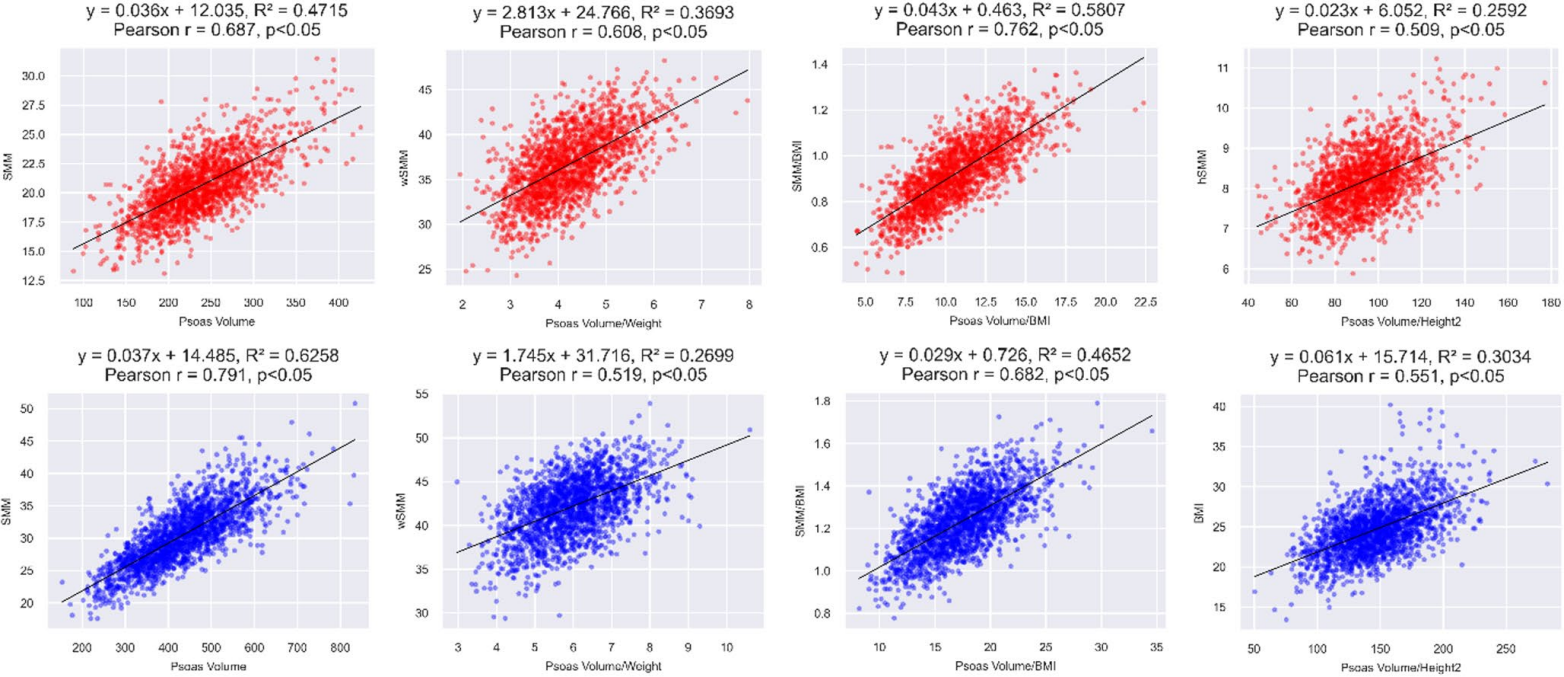
—Correlation between Psoas muscle volume indices and SMM indices (female - red, male - blue). SMM; Skeletal Muscle Mass, BMI; Body Mass Index

Figures 2 and 3 demonstrate the correlations between PV indices and ASM/SMM indices. In females, the correlations range from 0.468 to 0.757, with the strongest correlation observed in psoas muscle/BMI. For males, the correlations vary from 0.436 to 0.752, with the highest correlation seen in PV, followed by psoas muscle/height2. Across both genders, the psoas muscle/BMI index demonstrates a notably strong correlation.

Figures 3 illustrate the correlations between the PV indices and SMM indices. For females, the correlation coefficients with SMM indices span from 0.489 to 0.755, showing the highest correlation in the PV/BMI. In males, these coefficients range between 0.231 and 0.789, with the most significant correlation observed in the PV. This analysis underscores the strong correlation of PV/BMI and the PV across both genders.

Overall, our analysis revealed that the average Pearson correlation coefficients between both SM) and SMM in relation to various PV indices were generally high, with the notable exception of male SMM showing a lower correlation. Importantly, the comparison between the average correlation coefficients for ASM and SMM did not yield statistically significant differences, suggesting that both ASM and SMM exhibit a comparable degree of correlation strength with PV indices.

### Diagnosis criteria

#### Diagnostic criteria of PV index derived via correlation method with ASM index for sarcopenia

The established ASM Index [17–20], as shown in Table 2, was applied to the study participants, and the corresponding prevalence of sarcopenia was also documented.

**Table 2:** The prevalence of sarcopenia of the previous studies and our study for the aged population as indicated

Indices	Age of the participants	Gender	Prevalence according to the diagnostic criteria of ASM indices	Reference	Calculated prevalence according to the diagnostic criteria correlated with ASM indices	Calculated prevalence according to the diagnostic criteria by the ROC with ASM indices	Calculated prevalence according to the diagnostic criteria by T-score < -2.5	Calculated prevalence according to the diagnostic criteria by T-score < -2.0
PV	70–84	Male	**63.2%** (Average age 76.4 ± 3.9)	Kim et al. [21]	31.5% (Average age 76.6 ± 3.95)	17.6% (Average age 76.6 ± 3.95)	25.5% (Average age 76.6 ± 3.95)	**49.3%** (Average age 76.6 ± 3.95)
		Female	**80.1%** (Average age 75.4 ± 3.9)	Kim et al. [21]	**69.5%** (Average age 75.7 ± 3.77)	22.9% (Average age 75.7 ± 3.77)	9.9% (Average age 75.7 ± 3.77)	22.7% (Average age 75.7 ± 3.77)
PV/Weight	≥ 65	Male	**38.3%** (Average age 73.6 ± 7.6)	Kim et al. [22]	3.7% (Average age 74.6 ± 5.84)	5.2% (Average age 74.6 ± 5.84)	7.2% (Average age 74.6 ± 5.84)	**21.8%** (Average age 74.6 ± 5.84)
		Female	**62.6%** (Average age 72.5 ± 6.7)	Kim et al. [22]	21.9% (Average age 73.5 ± 5.47)	4.3% (Average age 73.5 ± 5.47)	8.7% (Average age 73.5 ± 5.47)	**25.8%** (Average age 73.5 ± 5.47)
PV/BMI	≥ 40	Male	**9.6%** (Average age 58.6 ± 10.7)	Kim et al. [19]	32.1% (Average age 60.9 ± 12.94)	5.8% (Average age 60.9 ± 12.94)	**7.9%** (Average age 60.9 ± 12.94)	17.2% (Average age 60.9 ± 12.94)
		Female	**7.9%** (Average age 58.5 ± 9.6)	Kim et al. [19]	31.0% (Average age 60.4 ± 12.23)	5.3% (Average age 60.4 ± 12.23)	**8.2%** (Average age 60.4 ± 12.23)	22.6% (Average age 60.4 ± 12.23)
PV/Height^2^	≥ 65	Male	**35.3%** (Average age 73.6 ± 7.6)	Kim et al. [22]	9.7% (Average age 74.3 ± 5.57)	10.2% (Average age 74.3 ± 5.57)	13.2% (Average age 74.3 ± 5.57)	**31.5%** (Average age 74.3 ± 5.57)
		Female	**13.4%** (Average age 72.5 ± 6.7)	Kim et al. [22]	**15.7%** (Average age 73.2 ± 5.18)	6.6% (Average age 73.2 ± 5.18)	2.6% (Average age 73.2 ± 5.18)	8.6% (Average age 73.2 ± 5.18)
	≥ 40	Male	**4.4%** (Male, Female Total Average 58.6 ± 10.7)	Kim et al. [19]	4.2% (Average age 60.9 ± 12.94)	**4.4%** (Average age 60.9 ± 12.94)	5.7% (Average age 60.9 ± 12.94)	14.8% (Average age 60.9 ± 12.94)
		Female	**0.4%** (Male, Female Total Average 58.5 ± 9.6)	Kim et al. [19]	8.7% (Average age 60.4 ± 12.23)	3.1% (Average age 60.4 ± 12.23)	**1.2%** (Average age 60.4 ± 12.23)	4.0% (Average age 60.4 ± 12.23)

ASM index pairs. ASM; Appendicular Skeletal Muscle, BMI; Body Mass Index. Note: In the table, bold text indicates the closest matching results between the two measurements

We focused on four pairs of ASM index-PV index, all in the same unit space. The cutoff value for the PV index was determined using a linear trend equation, considering the corresponding ASM index as the input (Table 1).

#### Diagnostic criteria of PV index obtained using ROC

We determined the cutoff values for PV indices using the ROC method. The ROC curve for the four PV-ASM index pairs is depicted, and the corresponding AUC is presented in Table 1. The cutoff values for PV indices were determined using the Youden cutoff at the point of maximum specificity with high sensitivity (Maximum Specificity + High Sensitivity Point), as summarized in Table 1.

#### Diagnostic criteria of PV index obtained using T-score

The values of each indicator corresponding to T-scores of -2.0 or below and − 2.5 or below, calculated using measurements from all participants, are presented in Table 1.

#### Comparison of the diagnostic criteria by three methods (correlation, ROC, and T-score)

The diagnostic criteria for the PV index and the corresponding prevalence rates, derived from the three analysis methods mentioned previously and benchmarked against established ASM diagnostic criteria [17–20], are presented in Table 1. To align the prevalence rates of sarcopenia with our findings, we recalibrated the ages of participants in our study to correspond with the age demographics of subjects in various referenced studies [19, 21–23], as detailed in Table 2.

## Discussion

This study revealed the distribution of PV in the general population, showing a clear decline in most indices from the 30s to 70s. Interestingly, the peak in PV appeared in the 30s for both men and women, not in the 20s, followed by a gradual decrease with age. In men, values increased from the 20s to peak in the 30s, while women showed no significant change between these decades. This suggests that age-related muscle mass change is smaller in women, possibly making differences too subtle to reach statistical significance.

Our findings align with other studies that have also confirmed different trends in muscle mass changes with age between genders [9, 24–27]. Previous research has shown that age-related muscle atrophy was greatest for the psoas major muscle among 10 muscles of the lower limb [28]. The psoas major muscle is considered to be closely related to locomotory capacity, such as running and stair climbing [29, 30]. Moreover, given the body of evidence suggesting that the psoas muscle effectively reflects sarcopenia, our study’s confirmation of the psoas muscle distribution in the general population stands as a significant contribution. This discovery highlights the psoas major muscle’s vital role not only in functional mobility but also in the context of age-related muscular health.

Prior research advocating the use of the psoas muscle for diagnosing sarcopenia has suggested that muscle mass analysis using CT scans could sufficiently replace current diagnostic methods such as DXA and BIA [31, 32]. However, a limitation of existing studies is that they often focus on specific patient groups, such as those with liver disease or cancer, using the PV for sarcopenia diagnosis [9, 33, 34]. This approach might be useful within these groups but has a small sample size and is disease-specific, limiting its representation of the general population. There are studies that have proposed criteria using the psoas muscle area at the L3 or L4 level for sarcopenia diagnosis [23–25]. The method utilizing the psoas muscle area has been criticized for its inter-observer variability and inconsistency in results, with the volume measurement method providing a more accurate representation of the entire muscle mass [10].

Another significant outcome of this study is the establishment of PV index criteria for diagnosing sarcopenia. We presented diagnostic criteria through three analytical methods. First, we used correlation analysis with the ASM index from the existing BIA method to validate our approach. The strong correlation between the psoas muscle index and ASM index supports PV as a reliable diagnostic criterion. The normal distribution and correlation of PV with ASM further support its feasibility.

Secondly, the ROC analysis we applied has been a longstanding method for establishing diagnostic criteria. This methodology has proven effective for defining new diagnostic benchmarks, such as in hepatocellular carcinoma diagnosis through abdominal ultrasound and alpha-fetoprotein testing [35], the integration of new pain biomarkers for lung consolidation diagnosis [36], and the establishment of criteria in vaccine phase I studies [37]. Similar to our study, there has been research employing the psoas muscle area and ROC analysis for diagnosing sarcopenia [38]. However, that analysis was limited to a small sample size of 82 individuals aged between 10 and 20 years and reported an AUC value of 0.697. In contrast, our study, which encompasses a much larger sample size of 3999 individuals, demonstrated AUC values ranging from 0.736 to 0.910, thereby bolstering confidence in our findings and the reliability of using PV indices as a diagnostic tool for sarcopenia.

In our study, the T-score was adopted as the third analytical method to establish diagnostic criteria for sarcopenia, mirroring the approach taken by the World Health Organization (WHO) for osteoporosis diagnosis. This T-score measures how an individual’s Bone Mineral Density deviates from that of a healthy young adult average, with a T-score of -2.5 or lower marking the threshold for osteoporosis according to WHO guidelines [39]. Notably, the risk of fracture approximately doubles for each standard deviation below the mean of a young adult’s BMD [40, 41]. Drawing a parallel, some studies have set the sarcopenia diagnostic threshold at 2 standard deviations below the mean, a method that, like its osteoporosis counterpart, is grounded in statistical analysis [42], but lacks the extensive clinical backing seen in osteoporosis diagnosis. In situations lacking extensive clinical data, we explored using T-scores of -2.5 and − 2.0 as thresholds to develop diagnostic criteria for sarcopenia. This approach, while drawing on statistical analysis similar to that used in osteoporosis diagnosis, aimed to establish a preliminary basis for sarcopenia diagnosis in the absence of robust clinical validation. The selection of an appropriate age range for normal reference values remains a contested area. Our findings indicate a decline in muscle volume from the age of 40 in both sexes, suggesting the 20s and 30s as a more suitable reference group for diagnosing sarcopenia. This is in harmony with osteoporosis diagnosis practices, which consider individuals under 50—around the age bone density typically begins to decline—as the baseline. Thus, our approach, which deems those under 40 as the norm, aligns with osteoporosis diagnostic standards, offering a logical and evidence-based framework for identifying sarcopenia.

The diagnostic criteria for sarcopenia, as suggested by three distinct analytical methods, vary, highlighting the need for additional studies to identify the optimal criteria for use. Given the established prevalence rates of sarcopenia, adjusting the T-score threshold to -2.0 from − 2.5, or employing calculations based on the correlation with the ASM index, resulted in a closer alignment of prevalence figures. This finding implies that such adjustments may warrant prioritization in future research and application.

Identifying the most appropriate index for diagnosing sarcopenia using PV demands a considered approach, similar to that employed with the ASM index in clinical practice. Our study demonstrates that the PV/BMI index yields the highest sarcopenia prevalence. High diagnostic accuracies within the PV indices in our study was achieved using the Pearson correlation coefficient along with prevalence rates, especially when considering the PV itself and PV/BMI. The selection of the best adjustment method for diagnosing sarcopenia, whether it be height, weight, or BMI, continues to stimulate debate within the field. Despite previous studies emphasizing the PV/height^2^ ratio for its strong correlation with the ASM index [43], our research offers new perspectives due to our expansive sample size and a comprehensive approach that extends beyond conventional height-based metrics. Furthermore, the Foundation for the National Institutes of Health Sarcopenia Project’s recommendation to use ASM/BMI as the diagnostic criterion for sarcopenia is supported by comprehensive population-based studies. This recommendation is backed by statistical analysis that directly links muscle mass index with indicators of physical frailty such as weakness and slowness. The significant correlation we observed between PV and ASM lends further credibility to the PV index as a robust marker for sarcopenia diagnosis, suggesting its effectiveness in clinical settings and its potential for broader application in sarcopenia assessment strategies.

Given the ongoing debate regarding the optimal body-size adjustment for muscle quantity (height²-, weight-, or BMI-adjusted indices), we interpreted our findings in the context of major diagnostic frameworks. EWGSOP/EWGSOP2 commonly reports low muscle quantity using height²-adjusted indices (e.g., ASM/height²), whereas the FNIH Sarcopenia Project proposed BMI-adjusted appendicular lean mass (ASM/BMI) as a clinically relevant criterion because it was empirically linked to weakness and slowness in large population-based datasets. In line with this BMI-adjusted approach, the PV/BMI index showed the strongest overall performance among the evaluated PV indices in our cohort (including correlation patterns and ROC-based diagnostic accuracy), suggesting that BMI adjustment may better account for body habitus when CT-derived psoas volume is used as a surrogate of muscle quantity. Nevertheless, because no single adjustment method is universally accepted and optimal indexing may differ across ethnicities and clinical settings, we present PV indices using multiple adjustment strategies and emphasize that external validation against functional measures and clinical outcomes is warranted.

While our study has limitations such as selection bias from a single health screening center and focus on the Korean population, it offers strengths including large sample size, precise automated measurements, and reproducible findings. We proposed CT-based PV index thresholds to identify low muscle quantity, using established ASM-based definitions as reference standards. Recognizing that strength loss results from both muscle mass reduction and factors like motor unit activation and muscle quality [44, 45], we suggest including muscle quality to improve diagnostic accuracy. Importantly, the proposed PV cut-offs should be interpreted as preliminary, as they were derived from a cross-sectional health-screening cohort without direct measures of muscle strength or physical performance. Therefore, prospective external validation is required against clinically relevant endpoints and functional criteria (e.g., handgrip strength and performance measures such as gait speed/SPPB) before these thresholds can be used to diagnose sarcopenia in clinical practice.

Our study highlights the value of opportunistic screening using existing CT scans, offering a novel, efficient approach to sarcopenia detection without requiring extra testing. This strategy streamlines diagnosis and care, potentially enhancing sarcopenia diagnostics and improving global patient outcomes.

## Conclusion

This study reveals the distribution of the volume of the psoas muscle across different age groups, emphasizing a notable decline from the 30s to the 70s. This advancement in objective diagnosis of sarcopenia via imaging positions abdomen CT scans as a potential diagnostic screening tool, laying the foundation for early detection and management of sarcopenia through timely interventions.

## Data Availability

No datasets were generated or analysed during the current study.

## Declarations

### Clinical Trial Number

Clinical trial number: not applicable.
